# Asymptomatic Hepatic Sequestration with Extreme Hyperbilirubinemia in an Adult Homozygous Sickle Cell Patient

**DOI:** 10.7759/cureus.7210

**Published:** 2020-03-08

**Authors:** Syed Musa Raza, Mehdi Faraji, Omar Khan, Rimsha Shaukat

**Affiliations:** 1 Internal Medicine, Louisiana State University Health Sciences Center, Shreveport, USA; 2 Radiology, Louisiana State University Health Sciences Center, Shreveport, USA

**Keywords:** sickle, hepatic, sequestration, hyperbilirubinemia

## Abstract

It has been estimated that there are greater than 100,000 individuals in the US with sickle cell disease. Hepatic sequestration is a known sequelae of sickle cell disease that rarely leads to extreme hyperbilirubinemia (total serum bilirubin greater than 20 mg/dl). Our 26-year-old male patient, though compliant with regular transfusion exchanges, presented with hepatic sequestration, with minimal symptoms and hyperbilirubinemia up to approximately 40 mg/dl. The severity of asymptomatic hepatic sequestration seen in our patient has never been reported in the literature. This mandates a low threshold to screen for sickle cell complications and promptly treating these patients admitted to the hospital with exchange transfusions.

## Introduction

Sickle cell disease complications are a common reason for hospital admissions. Acute hepatic manifestations are a subset of sickle cell disease admissions that account for somewhere around 10% of hospitalizations [1]. The spectrum of hepatic disease in sickle cell anemia varies from symptom control and self-resolution to the extreme of death from liver failure and hemorrhage due to loss of anticoagulation factors. Acute hepatic sequestration in sickle cell only requires two things: right upper quadrant abdominal pain and hepatomegaly [1]. Also, there can be associated nausea, vomiting, and jaundice. Hyperbilirubinemia greater than 20 mg/dl is required to make the diagnosis, but levels as high as 50-60 mg/dl are commonly seen in the pediatric population that recover with only supportive care [2]. Intravascular hemolytic breakdown increases the serum bilirubin which exceeds the livers clearance capacity [1]. On the contrary, in adult populations, it requires further symptom control interventions i.e. intravenous fluids and red blood cell transfusion exchange reactions due to the sickling of their red blood cells [2]. It has been hypothesized that the right upper quadrant pain results from stagnant red blood cells locally in the liver causing minimal circulation in hepatic sinusoids and resultant hypoxia [3]. The resulting hyperbilirubinemia is thought to be due to the breakdown of these static sickled red blood cells that flood the bile canaliculi and overload the local Kupffer cell capacity [1,3,4]. Overload of direct bilirubin compresses the biliary tree and backs up into the circulation [1,3,4]. The natural course of sickle cell patients includes a gradual worsening of their organ systems. In addition to non-compliance with chronic red blood cell replacement and/or chelation therapy, patients are at high risk of damaging their livers with an overload of iron, leading to their liver being damaged at an increasingly faster rate [1,3,5].

In the laboratory, other than hyperbilirubinemia, low hemoglobin/hematocrit, reticulocytosis, and minimal elevation in aspartate and alanine aminotransferase are also observed [5]. It should be noted that viral hepatitis would not usually present itself with such elevated bilirubin, and the aminotransferases would be more elevated and remain elevated for a longer duration of time [5]. Imaging modalities such as computed tomography and ultrasound will assist in diagnosing hepatomegaly [1,3,5]. Thus, the diagnosis of hepatic sequestration is a diagnosis that involves a combination of clinical, lab work, and imaging. The symptomatic and laboratory profile of the patient tends to improve after a partial exchange transfusion. The resolution of an acute hepatic sequestration crisis normally requires 3-4 days, demonstrated by an increase in the hematocrit. This increase in hematocrit is potentially dangerous and requires a watchful eye as hyperviscosity can increase the risk of heart failure, stroke, and even acute coronary syndrome, which would then benefit from phlebotomy [4,5]. It should be noted that a liver percutaneous biopsy is no longer recommended and has shown to lead to life-threatening hemorrhage and death in 28%-36% of cases as seen in United Kingdom patients [6-8].

## Case presentation

**Figure 1:**
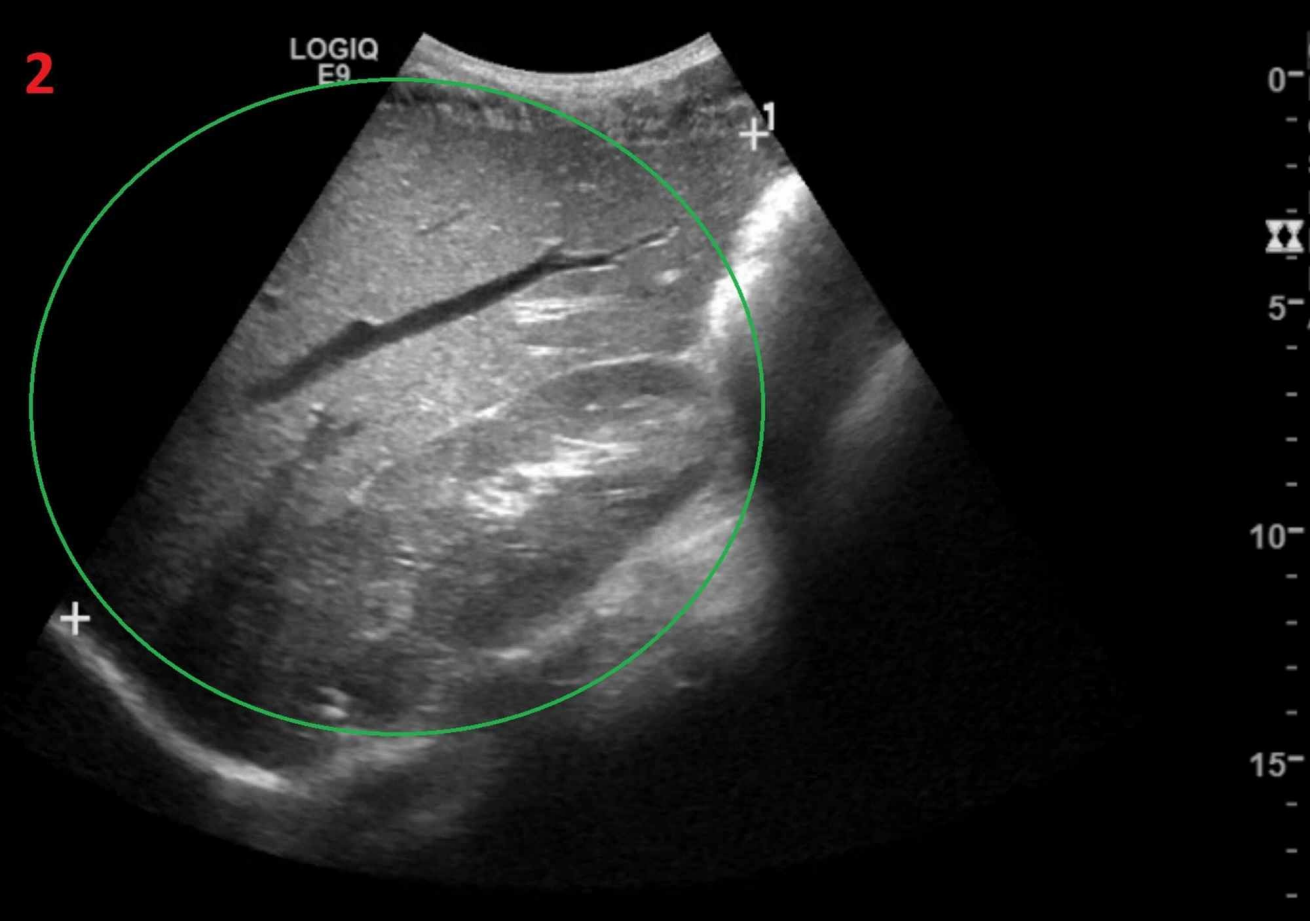
Ultrasound of the liver Enlarged liver seen (green circle)

We present a case of a 26-year-old African American male homozygous for sickle cell anemia, who underwent a cholecystectomy and splenectomy 20 years ago secondary to splenic sequestration. He presented to the clinic with concern of being “slightly jaundiced” for one day. After extensive research, we found that our case is unique in two aspects. No case has ever been documented for an asymptomatic patient with acute hepatic sequestration, or hyperbilirubinemia levels as high as 41 mg/dl in an adult patient. Only significant findings were mild jaundice with reticulocytosis, and hyperbilirubinemia of 39 mg/dl, of which 25 mg/dl was the direct bilirubin fraction. The abdominal ultrasound showed an enlarged liver measuring 18.2 cm (Figure 1).

**Figure 2:**
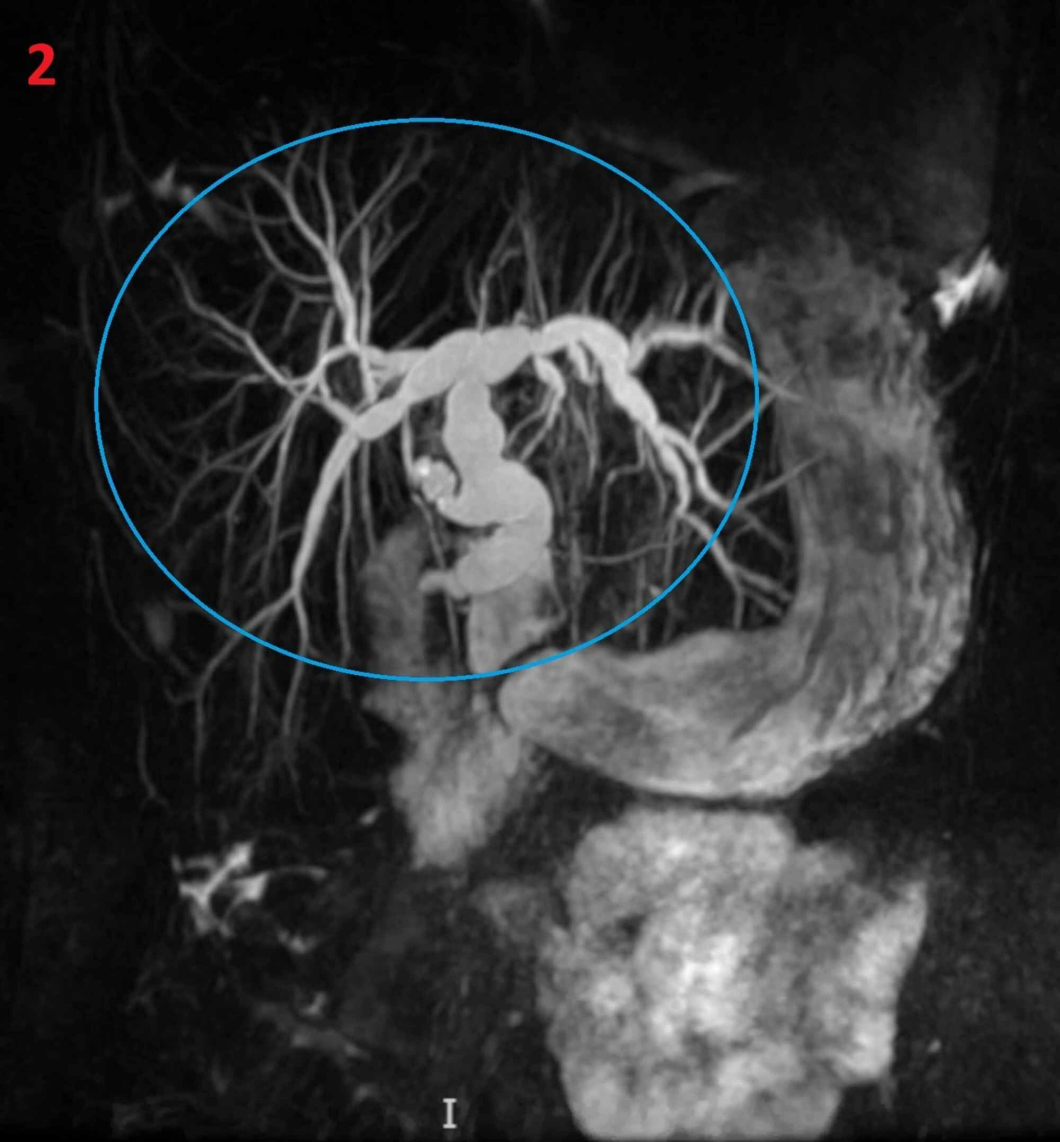
Magnetic resonance cholangiopancreatography (MRCP) MRCP showing intra and extrahepatic biliary ductal dilatation (blue circle)

The patient was then started on intravenous fluids, subsequently total bilirubin increased to 41 mg/dl, with direct bilirubin fraction of 35 mg/dl which then plateaued at 41 mg/dl. The patient’s hyperbilirubinemia continued to increase which prompted the initiation of a full exchange transfusion of his red blood cells on the third day of hospitalization. The patient’s total bilirubin decreased to 16.2, and he remained asymptomatic the following three days. Despite the above interventions, there was only a minimal amount of decrease in bilirubin levels, hence, we conducted a magnetic resonance cholangiopancreatography (MRCP). The study showed hepatomegaly and hypointensity of the hepatic parenchyma suggesting iron overload and noncompliance with his iron chelation medicine. Intra and extrahepatic biliary ductal dilatation were also noted on his MRCP, and gastroenterology was consulted (Figure 2).

It should be noted that magnetic resonance imaging (MRI) of the abdomen was done three years earlier and the patient did not show any biliary dilatation. Gastroenterology (GI) recommended an extensive workup that returned normal. This included the following labs: hepatitis B surface antibody, hepatitis B surface antigen, hepatitis B core antibody (immunoglobulin (Ig) M and IgG), hepatitis C antibody and reflex to polymerase chain reaction (PCR), hepatitis A IgM and IgG. Antinuclear antibody (ANA), human immunodeficiency virus (HIV), cytomegalovirus (CMV), herpes simplex virus (HSV), total Immunoglobulins levels, ceruloplasmin, alpha-1-antitrypsin, anti-mitochondrial antibody, anti-smooth muscle antibody, and anti-liver-kidney microsome antibody (LKMA) were all negative. Eventually, the patient’s total bilirubin decreased to 6.8 with a direct bilirubin fraction of 6. Upon discharge, the patient was educated on the importance of being compliant with his treatment and keeping a close follow up with hematology/oncology and gastroenterology.

## Discussion

This case was not only unique in that it involved extremely high bilirubin levels not commonly seen in adult patients with acute hepatic sequestration, but also because the patient was asymptomatic and adamant against admission and wanted to leave many times while he was admitted, requiring several conversations to persuade the patient to stay and continue being treated. It was with the help of radiological studies, ultrasound, and MRCP that we were able to see this patient’s enlarged liver and biliary dilatation. This case demonstrated to us and the medical community as a whole that asymptomatic patients can present in acute hepatic sequestration crisis, and that one’s threshold for conducting lab and imaging tests should be low, and not just based on the right upper quadrant abdominal pain that is classically expected on presentation [1,3-6]. This patient was treated symptomatically with continued elevation in his hyperbilirubinemia, which eventually necessitated a full red blood cell transfusion exchange. Had we not caught this early enough, it is hard to predict the outcome of his extreme hyperbiliruinemia and hepatic damage that would have continued to occur, especially with his history of iron chelation noncompliance.

It is important that the medical community as a whole and more specifically the gastroenterology and hematology/oncology communities are aware of the importance of imaging in asymptomatic sickle cell patients who present with only mild jaundice to prevent hepatic complications. This may entail routine MRCP on patients that present with jaundice, but at the very least bilirubin levels should be obtained [4,5,7]. To our knowledge, this is the first report that describes this unusual scenario with a favorable outcome.

## Conclusions

Sickle cell disease complications are a common cause of hospitalizations. Hepatic sequestration is one such complication that is not uncommon, but it has never been reported in adults at the level we saw in our patient. Our patient was a young asymptomatic male who presented with total serum bilirubin greater 40 mg/dl. Factoring in other studies, which include imaging like MRCP, to look for biliary dilatation and labs to look for elevated bilirubin levels allows for appropriate diagnosis, management, and treatment of an asymptomatic patient such as ours. This patient required admission for supportive treatment and a full red blood cell exchange transfusion. Hence, the appropriate management of this condition requires a collaborative effort involving clinical judgment, laboratory testing, and imaging.
